# Na_3_Co_2_(As_0.52_P_0.48_)O_4_(As_0.95_P_0.05_)_2_O_7_

**DOI:** 10.1107/S1600536813032029

**Published:** 2013-11-30

**Authors:** Youssef Ben Smida, Abderrahmen Guesmi, Mohamed Faouzi Zid, Ahmed Driss

**Affiliations:** a Laboratoire de Matériaux et Cristallochimie, Faculté des Sciences de Tunis, Université de Tunis ElManar, 2092 Manar II Tunis, Tunisia

## Abstract

The title compound, trisodium dicobalt(II) (arsenate/phosphate) (diarsenate/diphosphate), was prepared by a solid-state reaction. It is isostructural with Na_3_Co_2_AsO_4_As_2_O_7_. The framework shows the presence of Co*X*2_2_O_12_ (*X*2 is statistically disordered with As_0.95_P_0.05_) units formed by sharing corners between Co1O_6_ octa­hedra and *X*2_2_O_7_ groups. These units form layers perpendicular to [010]. Co2O_6_ octa­hedra and *X*1O_4_ (*X*1 = As_0.54_P_0.46_) tetra­hedra form Co2*X*1O_8_ chains parallel to [001]. Cohesion between layers and chains is ensured by the *X*2_2_O_7_ groups, giving rise to a three-dimensional framework with broad tunnels, running along the *a*- and *c*-axis directions, in which the Na^+^ ions reside. The two Co^2+^ cations, the *X*1 site and three of the seven O atoms lie on special positions, with site symmetries 2 and *m* for the Co, *m* for the *X*1, and 2 and *m* (× 2) for the O sites. One of two Na atoms is disordered over three special positions [occupancy ratios 0.877 (10):0.110 (13):0.066 (9)] and the other is in a general position with full occupancy. A comparison between structures such as K_2_CdP_2_O_7_, α-NaTiP_2_O_7_ and K_2_MoO_2_P_2_O_7_ is made. The proposed structural model is supported by charge-distribution (CHARDI) analysis and bond-valence-sum (BVS) calculations. The distortion of the coordination polyhedra is analyzed by means of the effective coordination number.

## Related literature

For isotypic structures, see: Ben Smail & Jouini (2005 ▶); Guesmi & Driss (2012 ▶). For related structures, see: Ben Smail & Jouini (2004 ▶); Boughzala *et al.* (1997 ▶); Faggiani & Calvo (1976 ▶); Leclaire *et al.* (1988 ▶); Geoffroy *et al.* (2011 ▶); Rissouli *et al.* (1996 ▶); Zid *et al.* (2003 ▶). For bond-valence analysis, see: Brown (2002 ▶); Adams (2003 ▶). For the charge distribution method, see: Nespolo *et al.* (2001 ▶); Nespolo (2001 ▶); Guesmi *et al.* (2006 ▶).

## Experimental

### 

#### Crystal data

Na_3_Co_2_(As_0.52_P_0.48_)O_4_(As_0.95_P_0.05_)_2_O_7_
*M* *_r_* = 562.32
Monoclinic, 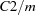

*a* = 10.3982 (9) Å
*b* = 16.087 (2) Å
*c* = 6.4421 (6) Å
β = 120.425 (9)°
*V* = 929.21 (17) Å^3^
*Z* = 4
Mo *K*α radiation
μ = 12.44 mm^−1^
*T* = 298 K
0.26 × 0.18 × 0.16 mm

#### Data collection

Enraf–Nonius CAD-4 diffractometer
Absorption correction: ψ scan (North *et al.*, 1968 ▶) *T*_min_ = 0.077, *T*_max_ = 0.138
2212 measured reflections
1052 independent reflections
974 reflections with *I* > 2σ(*I*)
*R*_int_ = 0.027
2 standard reflections every 120 reflections intensity decay: 1.4%

#### Refinement

*R*[*F* ^2^ > 2σ(*F* ^2^)] = 0.020
*wR*(*F* ^2^) = 0.049
*S* = 1.15
1052 reflections
105 parameters
3 restraints
Δρ_max_ = 0.61 e Å^−3^
Δρ_min_ = −0.61 e Å^−3^

### 

Data collection: *CAD-4 EXPRESS* (Duisenberg, 1992 ▶; Macíček & Yordanov, 1992 ▶); cell refinement: *CAD-4 EXPRESS*; data reduction: *XCAD4* (Harms & Wocadlo, 1995 ▶); program(s) used to solve structure: *SHELXS97* (Sheldrick, 2008 ▶); program(s) used to refine structure: *SHELXL97* (Sheldrick, 2008 ▶); molecular graphics: *DIAMOND* (Brandenburg & Putz, 2001 ▶); software used to prepare material for publication: *WinGX* (Farrugia, 2012 ▶) and *publCIF* (Westrip, 2010 ▶).

## Supplementary Material

Crystal structure: contains datablock(s) I. DOI: 10.1107/S1600536813032029/br2232sup1.cif

Structure factors: contains datablock(s) I. DOI: 10.1107/S1600536813032029/br2232Isup2.hkl

Additional supplementary materials:  crystallographic information; 3D view; checkCIF report

## Figures and Tables

**Fig. 1.**
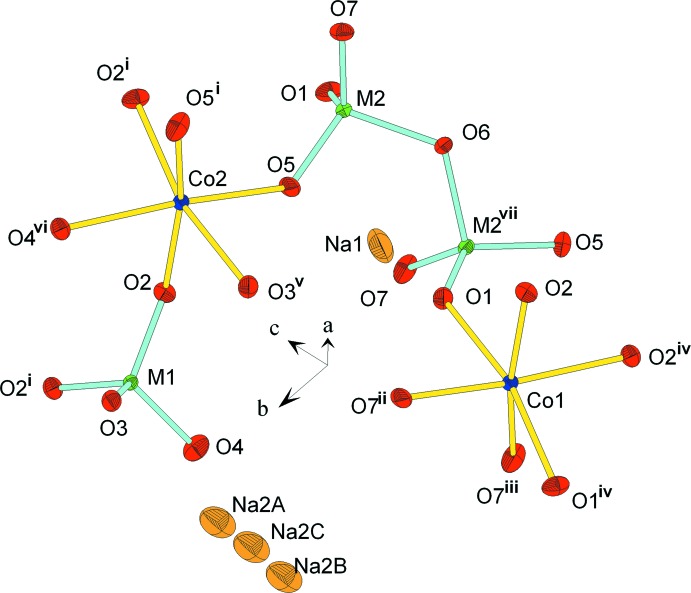
: L'unité formulaire dans Na3Co2(As0,52P0,48)O4(As0,95P0,05)2O7. Les ellipsoïdes sont définis avec 50% de probabilité. [codes de symétrie]: (i) x, -y + 1, z; (ii) x + 1/2, -y + 1/2, z; (iii) -x + 1/2, -y + 1/2, -z + 1; (iv) -x + 1, y, -z + 1; (v) -x + 1, -y + 1, -z + 1; (vi) -x + 1, -y + 1, -z + 2; (vii) -x, y, -z.

**Fig. 2.**
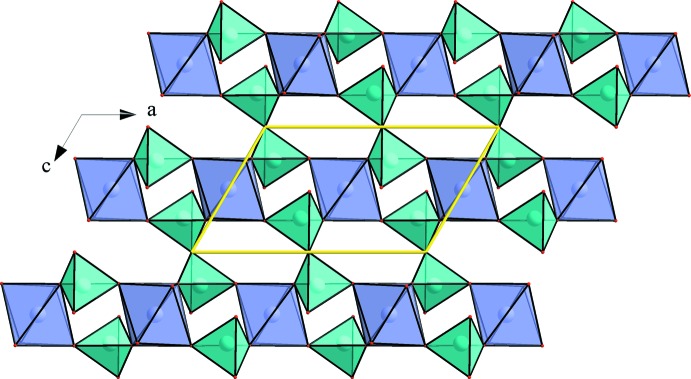
: Vue d'une couche selon la direction [010] montrant la jonction entre l'octraèdre Co1O6 et le groupement M22O7 (M2=As0,95P0,05).

**Fig. 3.**
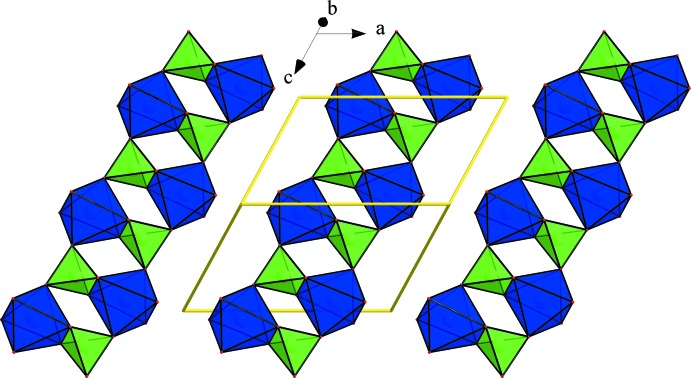
: Vue de rubans selon la direction [011] montrant la mise en commun d'arrête entre l'octraèdre Co2O6 et le tétraèdre M1O4 (M1=As0,52P0,48).

**Fig. 4.**
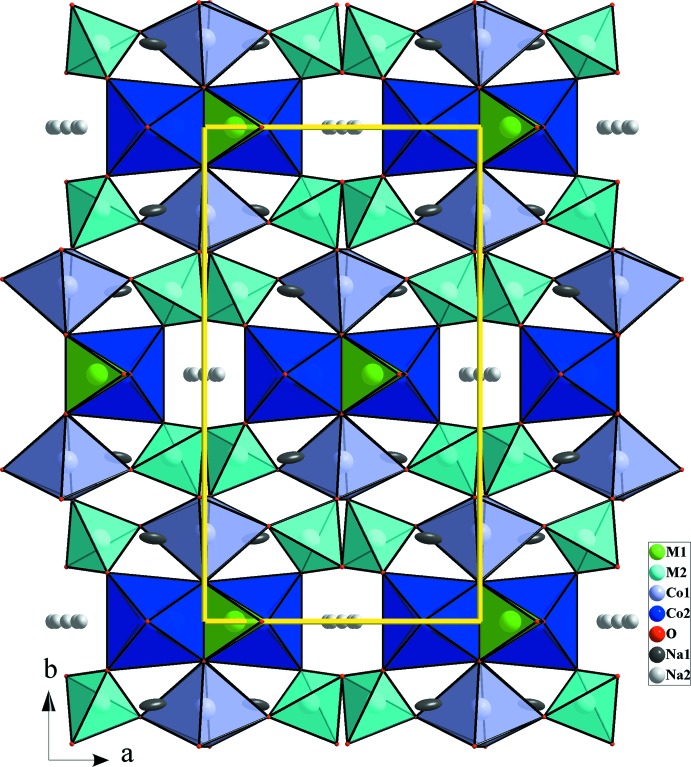
: Projection de la structure selon la direction [001].

**Fig. 5.**
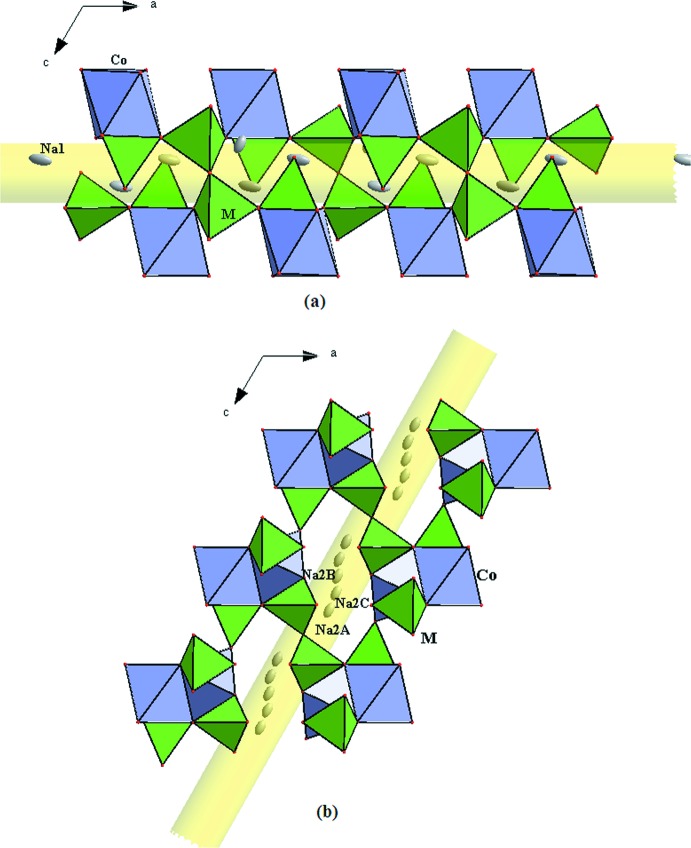
: Matérialisation de tunnels. (a): selon la direction [100]; (b): selon la direction [001].

## supplementary crystallographic information

### 1. Comment

La recherche de nouveaux matériaux à structures tridimensionnelles, ayant des larges tunnels utilisés pour des applications électrochimiques, constitue un domaine d'intense activité en chimie des solides. *L*'attention de plusieurs équipes s'est portée sur les phosphates doubles de métaux de transition et de cations monovalents présentant une stabilité thermique remarquable (Geoffroy *et al.*, 2011). Dans des travaux antérieurs, il a été montré qu'il existe une grande ressemblance structurale entre les phosphates et les arséniates ainsi que la possibilité d'une substitution de Phosphore par l'Arsenic et inversement (Ben Smail & Jouini, 2004, 2005; Boughzala *et al.*, 1997). Cependant la particularité structurale des arséniates vient de la possibilité d'avoir un environnement octaédrique chez l'Arsenic (Guesmi *et al.*, 2006) engendrant une richesse structurale à la famille des arséniates qui demeure peu étudiée.

Dans ce contexte, nous avons tenté d'explorer les systèmes A—Co—As/P—O (A=cation monovalent) par réaction à l'état solide. Cette investigation nous a permis d'isoler une nouvelle phase de formule Na_3_Co_2_(As_0,52_P_0,48_)O_4_(As_0,95_P_0,05_)_2_O_7_ isostructurale à Na_3_Co_2_AsO_4_As_2_O_7_ (Guesmi & Driss, 2012) et Na_3_Ni_2_(As_0,1_P_0,9_)O_4_(As_1,3_P_0,7_)O_7_ (Ben Smail & Jouini, 2005). *L*'unité formulaire de ce matériau (Fig. 1) est constituée de deux octaèdres CoO_6_, un tétraèdre M1O_4_ (M1=As_0,52_P_0,48_), un groupement M2_2_O_7_ (M2=As_0,95_P_0,05_) et trois atomes de sodium où l'un est statistiquement désordonné sur trois positions. *L*'examen de la charpente anionique révèle la présence de l'unité linéaire Co1M2_2_O_12_ constituée par un octaèdre Co1O_6_ partageant un seul sommet avec un groupement M2_2_O_7_ (Fig. 2). Ces unités se regroupent entre elles au moyen de ponts mixtes pour conduire à des couches disposées perpendiculairement à la direction [010] (Fig. 2). Les octaèdres Co2O_6_ et les tétraèdres M1O_4_ se lient par partage de sommets pour former des chaînes classiques Co2M1O_8_ orientées selon la direction [001] (Fig. 3). La disposition *cis* des tétraèdres M1O_4_ permet la jonction de ces chaînes par mise en commun d'arrêtes entre polyèdres de nature différente pour former un nouveau type de doubles chaînes de formulation Co2_2_M1_2_O_13_ (Fig. 3). La cohésion entre les couches et les doubles chaînes est assurée moyennant les groupements M2_2_O_7_ par mise en commun de sommets non engagés dans les couches. Il en résulte une structure tridimensionnelle (Fig. 4) ayant des larges tunnels disposés respectivement selon les directions [100] et [001] et des autres moins larges orientés selon la direction [110]. Les ions Na1 occupant une position générale et présentant un polyèdre déformé, apparaissent à l'intersection des tunnels disposés selon les directions [100] et [110] (Fig. 5a). Par contre les ions Na2 sont localisés à l'intersection des tunnels orientés selon les directions [001] et [110] (Fig. 5b).

La comparaison de la structure de Na_3_Co_2_(As_0,52_P_0,48_)O_4_(As_0,95_P_0,05_)_2_O_7_ avec des travaux antérieurs et renfermant principalement l'unité linéaire *MX*_2_O_12_ (*M* = métal bi ou trivalent, *X* = P, As) révèle une certaine filiation avec les matériaux K_2_CdP_2_O_7_, K_2_CaAs_2_O_7_ (Faggiani & Calvo, 1976) et α-NaTiP_2_O_7_ (Leclaire *et al.*, 1988). En effet, les unités *MX*_2_O_12_ se connectent par partage de sommets pour conduire à des couches qui à leurs tours se lient par ponts mixtes dans le trois directions formant ainsi des structures tridimensionnelles dans K_2_CdP_2_O_7_, K_2_CaAs_2_O_7_ et α-NaTiP_2_O_7_. Cependant dans les matériaux Li_2_Ni_3_(P_2_O_7_)_2_ (Rissouli *et al.*, 1996), K_2_MoO_2_P_2_O_7_ (Zid *et al.*, 2003) les groupements diphosphates forment avec les métaux des unités cycliques de type MP_2_O_11_. Ces dernières se connectent par partage des sommets entre polyèdres de nature differente pour conduire à des rubans formant une structure unidimensionnelle dans la phase K_2_MoO_2_P_2_O_7_. Dans la structure de Li_2_Ni_3_(P_2_O_7_)_2_, les unités cycliques de type NiP_2_O_11_ se connectent par arrêtes pour former des rubans qui se lient par partage de sommets dans le trois directions afin d'aboutir à une structure tridimentionnelle.

Le modèle structural proposé, particulièrement la distribution aux sites M1 et M2 et le désordre de l'atome de sodium Na2, est confirmé par les deux modèles de validation: la somme des valences de liaisons BVS (Brown, 2002; Adams, 2003) et la méthode de distribution de charge CHARDI (Nespolo *et al.*, 2001; Nespolo, 2001) (Table 1). Les valeurs de charges calculées Q(i) et de valences V(i) sont en bon accord avec les degrés d'oxydation pondérés par les taux d'occupation. Le facteur de dispersion σ_cat_ (Nespolo, 2001) qui mesure la déviation des charges cationiques calculées est égal à 6,4%. Pour les atomes d'oxygène, le facteur de dispersion σ_ana_ est égal à 11%. Un effet OUB (*Over-Under Bonding effect*) (Nespolo, 2001) est observé pour les deux atomes O2 et O4 avec des charges calculées respectivement égales à -1,829 et -2,190. Une explication de cet effet peut être attribuée à la mise en commun d'une arrête entre les polyèdres Co2O_6_ et M1O_4_ (Fig. 3). Ce type de jonction induit une forte répulsion cationique se repercutant sur les distances interatomiques et par conséquent sur les charges. Les polyèdres Co2O_6_ et M1O_4_ sont ainsi assez distordus avec des nombres de coordination effectifs respectivement égaux à ECoN(Co2)= 5,672 et ECoN(M1)= 3,966. Les distances, moyenne classique d_moy_ et arithmétique pondérée d_med_, sont cependant très proches et sont respectivement égales à 2,125 Å et 2,122 Å pour Co2 et 1,611 Å et 1,610 Å pour M1.

### 2. Experimental

La synthèse de la phase Na_3_Co_2_(As_0,52_P_0,48_)O_4_(As_0,95_P_0,05_)_2_O_7_ a été réalisée dans un creuset en porcelaine. Les réactifs, Na_2_CO_3_ (Prolabo, 27778), Co(NO_3_)_2_·6H_2_O (Fluka, 60832), NH_4_H_2_PO_4_ (Scharlau, 62943) et NH_4_H_2_AsO_4_ (préparé au laboratoire, ASTM 01–775), sont pris respectivement dans les rapports Na:Co:As:P égaux à 4:2:3:1. Le mélange finement broyé, est préchauffé lentement dans un four à moufle jusqu'à 623 K en vue d'éliminer NH_3_, H_2_O et NO_2_. Après refroidissement et un broyage poussé, le produit formé est porté jusqu'à une température (973 K) proche de la fusion. Il est alors abandonné à cette température pendant 3 jours pour favoriser la germination et la croissance des cristaux. Le résidu final a subi en premier lieu un refroidissement lent (5°/h) jusqu'à 923 K puis rapide (50°/h) jusqu'à la température ambiante. Des cristaux de couleur rose, de taille suffisante pour les mesures des intensités, ont été séparés du flux par l'eau bouillante.

### 3. Refinement

L'instruction SUMP est une contrainte linéaire autorisée par le programme d'affinement *SHELX*. Elle permet à deux atomes ou plus d'occuper le même site, avec la somme de taux d'occupation retenue comme une constante. Dans ce cas, avec l'instruction SUMP, on doit appliquer obligatoirement les contraintes: EXYZ et EADP. Ces dernières permettent d'attribuer aux atomes respectivement les mêmes coordonnées et facteurs d'agitation thermique.

L'utilization des instructions SUMP et EADP pour les couples d'ions As1/P1, As2/P2 et Na2A/Na2B/Na2C conduit à des ellipsoïdes bien définis. De plus, les densités d'électrons maximum et minimum restants dans la Fourier-différence sont acceptables et sont situées respectivements à 0,77 Å de Na1 et à 0,37 Å de Co2.

### Crystal data

**Table:**

As_2.42_Co_2_Na_3_O_11_P_0.57_	*F*(000) = 1055
*M**_r_* = 562.32	*D*_x_ = 4.020 Mg m^−^^3^
Monoclinic, *C*2/*m*	Mo *K*α radiation, λ = 0.71073 Å
Hall symbol: -C 2y	Cell parameters from 25 reflections
*a* = 10.3982 (9) Å	θ = 10–15°
*b* = 16.087 (2) Å	µ = 12.44 mm^−^^1^
*c* = 6.4421 (6) Å	*T* = 298 K
β = 120.425 (9)°	Prism, pink
*V* = 929.21 (17) Å^3^	0.26 × 0.18 × 0.16 mm
*Z* = 4	

### Data collection

**Table:**

Enraf–Nonius CAD-4 diffractometer	974 reflections with *I* > 2σ(*I*)
Radiation source: fine-focus sealed tube	*R*_int_ = 0.027
Graphite monochromator	θ_max_ = 27.0°, θ_min_ = 2.5°
ω/2θ scans	*h* = −13→13
Absorption correction: ψ scan (North *et al.*, 1968)	*k* = −20→1
*T*_min_ = 0.077, *T*_max_ = 0.138	*l* = −8→8
2212 measured reflections	2 standard reflections every 120 reflections
1052 independent reflections	intensity decay: 1.4%

### Refinement

**Table:**

Refinement on *F*^2^	Primary atom site location: structure-invariant direct methods
Least-squares matrix: full	Secondary atom site location: difference Fourier map
*R*[*F*^2^ > 2σ(*F*^2^)] = 0.020	*w* = 1/[σ^2^(*F*_o_^2^) + (0.012*P*)^2^ + 5.1693*P*] where *P* = (*F*_o_^2^ + 2*F*_c_^2^)/3
*wR*(*F*^2^) = 0.049	(Δ/σ)_max_ < 0.001
*S* = 1.15	Δρ_max_ = 0.61 e Å^−^^3^
1052 reflections	Δρ_min_ = −0.61 e Å^−^^3^
105 parameters	Extinction correction: *SHELXL*, Fc^*^=kFc[1+0.001xFc^2^λ^3^/sin(2θ)]^-1/4^
3 restraints	Extinction coefficient: 0.00084 (13)

### Special details

**Table:**

Geometry. All e.s.d.'s (except the e.s.d. in the dihedral angle between two l.s. planes) are estimated using the full covariance matrix. The cell e.s.d.'s are taken into account individually in the estimation of e.s.d.'s in distances, angles and torsion angles; correlations between e.s.d.'s in cell parameters are only used when they are defined by crystal symmetry. An approximate (isotropic) treatment of cell e.s.d.'s is used for estimating e.s.d.'s involving l.s. planes.
Refinement. Refinement of *F*^2^ against ALL reflections. The weighted *R*-factor *wR* and goodness of fit *S* are based on *F*^2^, conventional *R*-factors *R* are based on *F*, with *F* set to zero for negative *F*^2^. The threshold expression of *F*^2^ > σ(*F*^2^) is used only for calculating *R*-factors(gt) *etc*. and is not relevant to the choice of reflections for refinement. *R*-factors based on *F*^2^ are statistically about twice as large as those based on *F*, and *R*- factors based on ALL data will be even larger.

### Fractional atomic coordinates and isotropic or equivalent isotropic displacement parameters (Å^2^)

**Table:**

	*x*	*y*	*z*	*U*_iso_*/*U*_eq_	Occ. (<1)
As1	0.61047 (7)	0.5000	0.82497 (11)	0.0066 (2)	0.519 (5)
P1	0.61047 (7)	0.5000	0.82497 (11)	0.0066 (2)	0.481 (6)
As2	0.11976 (4)	0.33459 (2)	0.26883 (6)	0.00837 (13)	0.953 (4)
P2	0.11976 (4)	0.33459 (2)	0.26883 (6)	0.00837 (13)	0.047 (4)
Co1	0.5000	0.32278 (4)	0.5000	0.00767 (18)	
Co2	0.30246 (7)	0.5000	0.58620 (11)	0.00979 (18)	
O1	0.2677 (3)	0.30726 (17)	0.2518 (4)	0.0159 (5)	
O2	0.4973 (3)	0.42032 (15)	0.7288 (4)	0.0125 (5)	
O3	0.7294 (4)	0.5000	0.7420 (6)	0.0130 (7)	
O4	0.7069 (4)	0.5000	1.1130 (6)	0.0175 (8)	
O5	0.1483 (3)	0.40165 (15)	0.4838 (4)	0.0137 (5)	
O6	0.0000	0.3895 (2)	0.0000	0.0126 (7)	
O7	0.0195 (3)	0.25247 (17)	0.2544 (4)	0.0204 (6)	
Na1	0.1865 (3)	0.16850 (11)	0.0966 (3)	0.0443 (6)	
Na2A	0.0524 (3)	0.5000	0.7917 (8)	0.0361 (12)	0.871 (10)
Na2B	0.0000	0.5000	0.5000	0.0361 (12)	0.111 (12)
Na2C	0.038 (4)	0.5000	0.643 (13)	0.0361 (12)	0.071 (9)

### Atomic displacement parameters (Å^2^)

**Table:**

	*U*^11^	*U*^22^	*U*^33^	*U*^12^	*U*^13^	*U*^23^
As1	0.0072 (3)	0.0073 (4)	0.0058 (3)	0.000	0.0037 (3)	0.000
P1	0.0072 (3)	0.0073 (4)	0.0058 (3)	0.000	0.0037 (3)	0.000
As2	0.00761 (19)	0.0082 (2)	0.00724 (19)	−0.00078 (13)	0.00228 (14)	−0.00001 (13)
P2	0.00761 (19)	0.0082 (2)	0.00724 (19)	−0.00078 (13)	0.00228 (14)	−0.00001 (13)
Co1	0.0072 (3)	0.0082 (3)	0.0074 (3)	0.000	0.0036 (2)	0.000
Co2	0.0096 (3)	0.0113 (3)	0.0094 (3)	0.000	0.0055 (3)	0.000
O1	0.0096 (11)	0.0173 (13)	0.0166 (12)	0.0020 (10)	0.0035 (10)	−0.0054 (10)
O2	0.0133 (11)	0.0115 (12)	0.0147 (11)	−0.0007 (10)	0.0086 (10)	−0.0013 (10)
O3	0.0176 (17)	0.0123 (17)	0.0101 (16)	0.000	0.0078 (14)	0.000
O4	0.0177 (18)	0.027 (2)	0.0113 (17)	0.000	0.0101 (15)	0.000
O5	0.0154 (12)	0.0144 (12)	0.0121 (11)	−0.0043 (10)	0.0075 (10)	−0.0042 (10)
O6	0.0122 (15)	0.0100 (16)	0.0091 (14)	0.000	0.0008 (13)	0.000
O7	0.0276 (14)	0.0179 (13)	0.0121 (12)	−0.0130 (12)	0.0074 (11)	0.0016 (11)
Na1	0.0860 (17)	0.0175 (9)	0.0239 (9)	0.0096 (10)	0.0238 (10)	−0.0010 (7)
Na2A	0.0202 (14)	0.0243 (15)	0.054 (3)	0.000	0.0117 (15)	0.000
Na2B	0.0202 (14)	0.0243 (15)	0.054 (3)	0.000	0.0117 (15)	0.000
Na2C	0.0202 (14)	0.0243 (15)	0.054 (3)	0.000	0.0117 (15)	0.000

### Geometric parameters (Å, º)

**Table:**

As1—O3	1.577 (4)	Na1—O1^vii^	2.549 (3)
As1—O4	1.601 (3)	Na1—O7^viii^	2.573 (3)
As1—O2	1.635 (2)	Na1—O2^ix^	2.609 (3)
As1—O2^i^	1.635 (2)	Na1—O5^iii^	2.613 (3)
As2—O1	1.656 (2)	Na1—O4^x^	2.717 (2)
As2—O7	1.656 (3)	Na2A—O4^vi^	2.255 (5)
As2—O5	1.659 (2)	Na2A—O6^xi^	2.449 (4)
As2—O6	1.773 (2)	Na2A—O6^xii^	2.449 (4)
Co1—O7^ii^	2.082 (2)	Na2A—O5^xiii^	2.501 (4)
Co1—O7^iii^	2.082 (2)	Na2A—O5^xii^	2.501 (4)
Co1—O1	2.131 (2)	Na2A—O3^vi^	2.696 (5)
Co1—O1^iv^	2.131 (2)	Na2B—O5^xiii^	2.249 (2)
Co1—O2	2.163 (2)	Na2B—O5^i^	2.249 (2)
Co1—O2^iv^	2.163 (2)	Na2B—O5^xii^	2.249 (2)
Co2—O3^v^	1.967 (3)	Na2B—O4^vi^	2.794 (4)
Co2—O4^vi^	1.989 (4)	Na2B—O4^xiv^	2.794 (4)
Co2—O5	2.106 (2)	Na2C—O4^vi^	2.30 (4)
Co2—O5^i^	2.106 (2)	Na2C—O5^xiii^	2.31 (3)
Co2—O2^i^	2.169 (2)	Na2C—O5^xii^	2.31 (3)
Co2—O2	2.169 (2)	Na2C—O5^i^	2.46 (5)
			
O3—As1—O4	104.73 (18)	O1^iv^—Co1—O2	88.54 (10)
O3—As1—O2	114.16 (11)	O7^ii^—Co1—O2^iv^	82.19 (10)
O4—As1—O2	110.33 (11)	O7^iii^—Co1—O2^iv^	168.43 (10)
O3—As1—O2^i^	114.16 (11)	O1—Co1—O2^iv^	88.54 (10)
O4—As1—O2^i^	110.33 (11)	O1^iv^—Co1—O2^iv^	101.27 (9)
O2—As1—O2^i^	103.25 (17)	O2—Co1—O2^iv^	86.97 (13)
O1—As2—O7	111.36 (14)	O3^v^—Co2—O4^vi^	169.21 (16)
O1—As2—O5	116.62 (12)	O3^v^—Co2—O5	88.62 (9)
O7—As2—O5	114.01 (13)	O4^vi^—Co2—O5	84.28 (10)
O1—As2—O6	106.49 (9)	O3^v^—Co2—O5^i^	88.62 (9)
O7—As2—O6	103.40 (12)	O4^vi^—Co2—O5^i^	84.28 (10)
O5—As2—O6	103.39 (12)	O5—Co2—O5^i^	97.39 (14)
O7^ii^—Co1—O7^iii^	108.88 (16)	O3^v^—Co2—O2^i^	93.91 (11)
O7^ii^—Co1—O1	82.59 (10)	O4^vi^—Co2—O2^i^	94.79 (11)
O7^iii^—Co1—O1	89.58 (10)	O5—Co2—O2^i^	167.36 (10)
O7^ii^—Co1—O1^iv^	89.58 (10)	O5^i^—Co2—O2^i^	95.05 (9)
O7^iii^—Co1—O1^iv^	82.59 (10)	O3^v^—Co2—O2	93.91 (11)
O1—Co1—O1^iv^	166.54 (15)	O4^vi^—Co2—O2	94.79 (11)
O7^ii^—Co1—O2	168.43 (10)	O5—Co2—O2	95.05 (9)
O7^iii^—Co1—O2	82.19 (10)	O5^i^—Co2—O2	167.36 (10)
O1—Co1—O2	101.27 (9)	O2^i^—Co2—O2	72.44 (13)

Symmetry codes: (i) *x*, −*y*+1, *z*; (ii) *x*+1/2, −*y*+1/2, *z*; (iii) −*x*+1/2, −*y*+1/2, −*z*+1; (iv) −*x*+1, *y*, −*z*+1; (v) −*x*+1, −*y*+1, −*z*+1; (vi) −*x*+1, −*y*+1, −*z*+2; (vii) −*x*+1/2, −*y*+1/2, −*z*; (viii) −*x*, *y*, −*z*; (ix) *x*−1/2, −*y*+1/2, *z*−1; (x) *x*−1/2, *y*−1/2, *z*−1; (xi) *x*, *y*, *z*+1; (xii) −*x*, −*y*+1, −*z*+1; (xiii) −*x*, *y*, −*z*+1; (xiv) *x*−1, *y*, *z*−1.

### Analyses CHARDI et BVS relatives aux cations dans Na_3_Co_2_(As_0,52_P_0,48_)O_4_(As_0,95_P_0,05_)_2_O_7_

**Table:**

Cation	q(i).sof(i)	V(i).sof(i)	Q(i)	CN(i)	ECoN(i)	d_moy_(i)	d_med_(i)
M1	5,000	5,131	5,146	4	3,966	1,611	1,610
M2	5,000	4,967	4,927	4	3,884	1,686	1,677
Co1	2,000	1,945	2,030	6	5,948	2,125	2,122
Co2	2,000	2,152	2,022	6	5,672	2,084	2,164
Na1	1,000	0,965	0,989	7	6,571	2,606	2,577
Na2*A*	0,882	0,969	0,846	6	5,412	2,475	2,430
Na2*B*	0,056	0,067	0,055	6	4,374	2,430	2,268
Na2*C*	0,071	0,086	0,069	5	4,817	2,366	2,351

q(i) = nombre d'oxydation formel; sof(i) = taux d'occupation du site; CN(i) = nombre de coordination classique ;Q(i)=charge calculée ;V(i)= valence calculée ; ECoN(i)= nombre de coordination effectif ; d_moy_(i) = distance arithmétique moyenne; d_med_(i) = distance pondérée moyenne; σ_cat_ = facteur de dispersion sur les charges cationiques ; σ_cat_ = [Σ_i_(q_i_-Q_i_)^2^/N-1]^1/2^=0,064.

## References

[bb1] Adams, S. (2003). *softBV* University of Göttingen, Germany. http://kristall.uni-mki.gwdg.de/softBV/

[bb2] Ben Smail, R. & Jouini, T. (2004). *Acta Cryst.* E**60**, i1–i2.

[bb3] Ben Smail, R. & Jouini, T. (2005). *Anal. Chem.* **30**, 119–132.

[bb4] Boughzala, H., Driss, A. & Jouini, T. (1997). *Acta Cryst.* C**53**, 3–5.

[bb5] Brandenburg, K. & Putz, H. (2001). *DIAMOND* Crystal Impact GbR, Bonn, Germany.

[bb6] Brown, I. D. (2002). *The Chemical Bond in Inorganic Chemistry - The Bond Valence Model* IUCr Monographs on Crystallography, No. 12. Oxford University Press.

[bb7] Duisenberg, A. J. M. (1992). *J. Appl. Cryst.* **25**, 92–96.

[bb8] Faggiani, R. & Calvo, C. (1976). *Can. J. Chem.* **54**, 3319–3324.

[bb9] Farrugia, L. J. (2012). *J. Appl. Cryst.* **45**, 849–854.

[bb10] Geoffroy, H., Anubhav, J., Shyue Ping, O., Byoungwoo, K., Charles, M., Robert, D. & Gerbrand, C. (2011). *Chem. Mater.* **23**, 3495–3508.

[bb11] Guesmi, A. & Driss, A. (2012). *Acta Cryst.* E**68**, i58. 10.1107/S1600536812027791 PMC3393142 22807699

[bb12] Guesmi, A., Nespolo, M. & Driss, A. (2006). *J. Solid State Chem.* **179**, 2466–2471.

[bb13] Harms, K. & Wocadlo, S. (1995). *XCAD4* University of Marburg, Germany.

[bb14] Leclaire, A., Benmoussa, A., Borel, M. M., Grandin, A. & Raveau, B. (1988). *J. Solid State Chem.* **77**, 299–305.

[bb15] Macíček, J. & Yordanov, A. (1992). *J. Appl. Cryst.* **25**, 73–80.

[bb16] Nespolo, M. (2001). *CHARDI-IT, CRM^2^* University Henri Poincaré Nancy I, France.

[bb17] Nespolo, M., Ferraris, G., Ivaldi, G. & Hoppe, R. (2001). *Acta Cryst.* B**57**, 652–664. 10.1107/s0108768101009879 11574721

[bb18] North, A. C. T., Phillips, D. C. & Mathews, F. S. (1968). *Acta Cryst.* A**24**, 351–359.

[bb19] Rissouli, K., Benkhouja, K., Sadel, A., Bettach, M., Zahir, M., Giorgi, M. & Pierrot, M. (1996). *Acta Cryst.* C**52**, 2960–2963.

[bb20] Sheldrick, G. M. (2008). *Acta Cryst.* A**64**, 112–122. 10.1107/S0108767307043930 18156677

[bb21] Westrip, S. P. (2010). *J. Appl. Cryst.* **43**, 920–925.

[bb22] Zid, M. F., Driss, A. & Jouini, T. (2003). *Acta Cryst.* E**59**, i65–i67.

